# Interrelation of the stagnant slab, Ontong Java Plateau, and intraplate volcanism as inferred from seismic tomography

**DOI:** 10.1038/s41598-021-99833-5

**Published:** 2021-10-28

**Authors:** Masayuki Obayashi, Junko Yoshimitsu, Daisuke Suetsugu, Hajime Shiobara, Hiroko Sugioka, Aki Ito, Takehi Isse, Yasushi Ishihara, Satoru Tanaka, Takashi Tonegawa

**Affiliations:** 1 Research Institute for Marine Geodynamics, Japan Agency for Marine-Earth Science and Technology, 2-15 Natsushimacho, Yokosuka, 237-0061 Japan; 2 Earthquake Research Institute, The University of Tokyo, Tokyo, 113-0032 Japan; 3 Graduate School of Science, Kobe University, Kobe, 657-8501 Japan

**Keywords:** Geodynamics, Geology, Geophysics, Seismology, Tectonics

## Abstract

We investigated the seismological structure beneath the equatorial Melanesian region, where is tectonically unique because an immense oceanic plateau, a volcanic chain and subduction zones meet. We conducted a multi-frequency P-wave tomography using data collected from an approximately 2-year-long seismic experiment around the Ontong Java Plateau (OJP). High-velocity anomalies were revealed beneath the center of the OJP at a depth of ~ 150 km, the middle-eastern edge of the OJP at depths of 200–300 km, and in the mantle transition zone beneath and around the OJP; low-velocity anomalies were observed along the Caroline volcanic island chain above 450 km depth. These anomalies are considered to be associated with the thick lithosphere of the OJP, remnant dipping Pacific slab, stagnant Pacific slab, and a sheet-like upwelling. The broad stagnant slab was formed due to rapid trench retreat from 48 to 25 Ma until when the OJP with thick lithosphere collided with a subduction boundary of the Pacific and Australian plates. This collision triggered slab breakoff beneath the arc where the dipping slab remained. The stagnant Pacific slab in the mantle transition zone restricted the plume upwelling from the lower mantle causing sheet-like deformed upwelling in the upper mantle.

## Introduction

The equatorial Melanesian region is characterized by intense tectonic activities: subduction zones along Papua New Guinea, Solomon Islands, and Vanuatu islands to the south, the Ontong Java Plateau (OJP) near the equator, and Caroline volcanic chain to the north. OJP, the largest and most voluminous of large igneous provinces, was initially formed at ~ 122 Ma at equatorial to mid southern latitudes in the Pacific Basin and moved approximately northeastward according to the Pacific plate motion^1,2^. The crustal thickness of the OJP is 30–40 km, which is comparable to that of continental crusts^3–6^. Beneath the crust, the seismic property of the “root” of the OJP is controversial. Richardson et al.^7^ revealed an OJP root with low S-wave velocity extending as deep as 300 km, whereas Covellone et al.^8^ and Isse et al.^9^ indicated high S-wave velocities beneath the central OJP down to a depth of 100 and 130 km, respectively. At the Solomon island arc located toward the southwest of the OJP, subduction of the plate switched from being south-directed to being north-directed, as the Australian plate began to subduct beneath the formerly consumed Pacific plate^10–12^. This reverse of the subduction polarity was initiated at 12–6 Ma due to the collision of the OJP with the subduction boundary at 25–10 Ma^12–14^. The subduction boundary at 45 Ma was located to the southwest of the current boundary and rapidly retreated until 25 Ma^15,16^. The retreat of the subduction boundary resulted in the deformation of the subducted slabs causing them to lie horizontally in the mantle transition zone^17–19^. Hall and Spakman^20^ interpreted broad high P-wave velocity anomalies at depths of 500–700 km extending from the Solomon island arc to the coral sea off the northeast coast of Australia as the subducted Pacific slab lying horizontally due to the rapid trench retreat.

**Figure 1:**
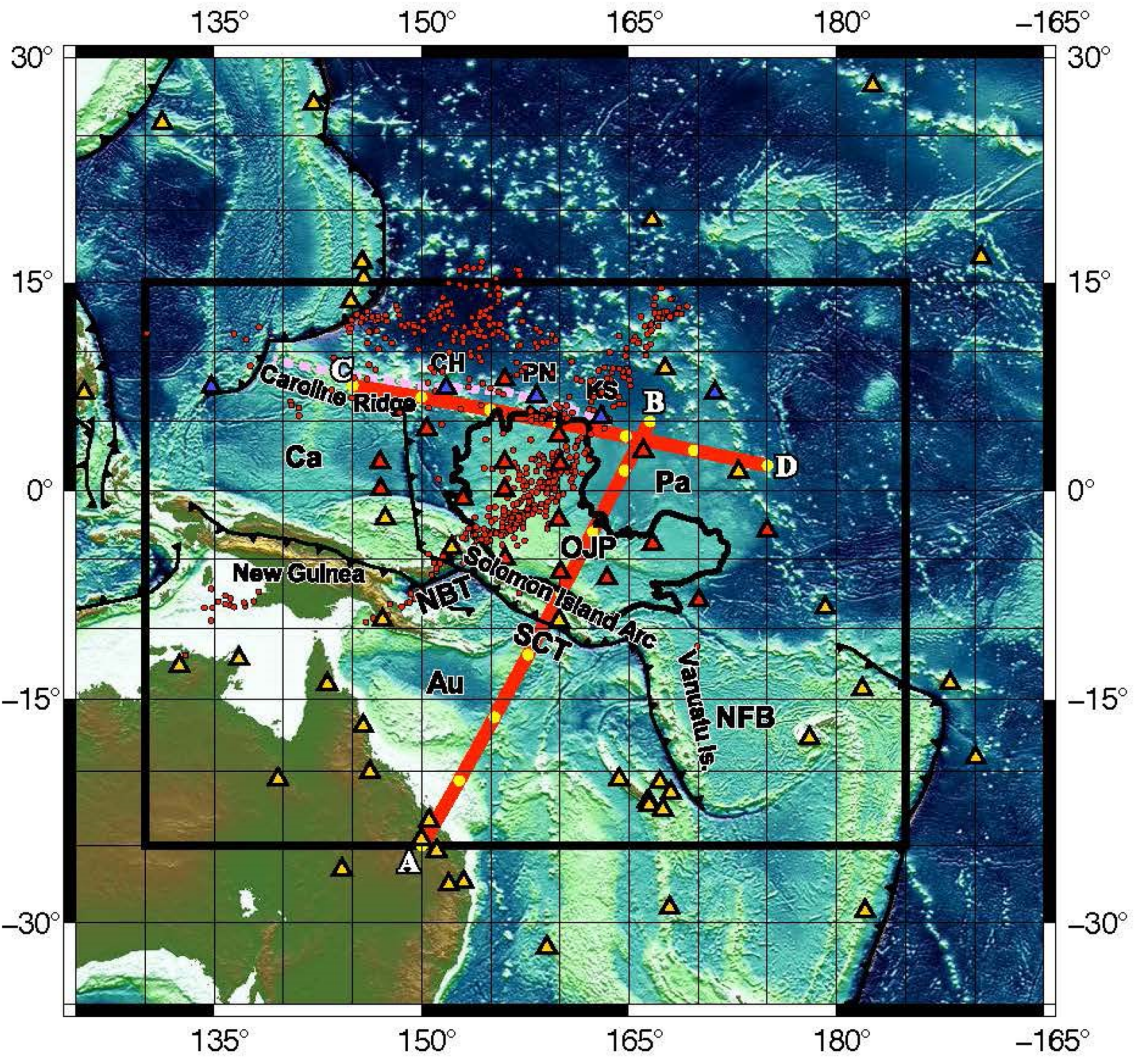
Stations used in this study to measure differential traveltimes between two stations as the function of frequency. Red and blue triangles indicate the broadband ocean bottom seismometers and island stations of the OJP array, respectively. The island stations operated by the Pacfic21 network are represented by blue triangles and the stations from IRIS data management center by yellow triangles. Red points indicate bounce points of the PP phase of which traveltime differences from the P phase are used for the inversion. Violet dotted line indicates the track of the Caroline hot spot^41^. Profiles of the cross sections in Fig. 2 are shown by red lines AB and CD with yellow dots at intervals of 5° from A and C, respectively. *Pa* Pacific plate, *Au* Australia plate, *Ca* Caroline plate, *OJP* Ontong Java Plateau, *CH* Chuuk, *PN* Ponape, *KS* Kosrae, *NBT* New Britain Trench, *SCT* San Cristobal Trench, and *NFB* North Fiji Basin. The Generic Mapping Tools (GMT) 5.4.5^53^ was used to make this figure.

The chain of the Caroline Islands of Chuuk, Ponape, and Kosrae (CH, PN, and KS, respectively in Fig. 1) at the northern flanks of the OJP indicates a progressive increase in age towards the west and is considered to be a volcanic hotspot^21–23^. The ages of lavas from individual islands vary widely (Chuuk, 12.7–4.7 Ma; Ponape, 8.7–0.9; Kosrae, 2.6–1.2 Ma), whereas those of other hotspot volcanoes in the Pacific do not exceed 3 Ma^24^. Wide time spans of lavas from Chuuk and Ponape and the overlapping ages of the Caroline Islands imply a complex hotspot process.

So far, the associations between the abovementioned tectonic features in the Melanesian region are poorly understood, mainly because the mantle structure beneath the region has not been explored owing to a lack of regional seismic data. Previous seismic tomography studies^20,25^ could not resolve the mantle down to 1000 km beneath the OJP due to the lack of the ray paths traveling through the region. Therefore, it has not been possible to discuss the relation between the surface tectonics and the deep structure. In this study, we developed a novel three-dimensional (3D) P-wave velocity structure by using data from a seismological experiment deployed in and around the OJP from late 2014 to early 2017^26^ and we succeeded in resolving the P-wave velocity structure in the upper mantle including the mantle transition zone. Through the results of this study, we illustrate the extended high-velocity anomalies in the mantle transition zone beneath the OJP and the sheet-like low velocity anomalies along the Caroline Islands, and discuss their relation and impacts on the surface tectonics.

## Results

**Figure 2:**
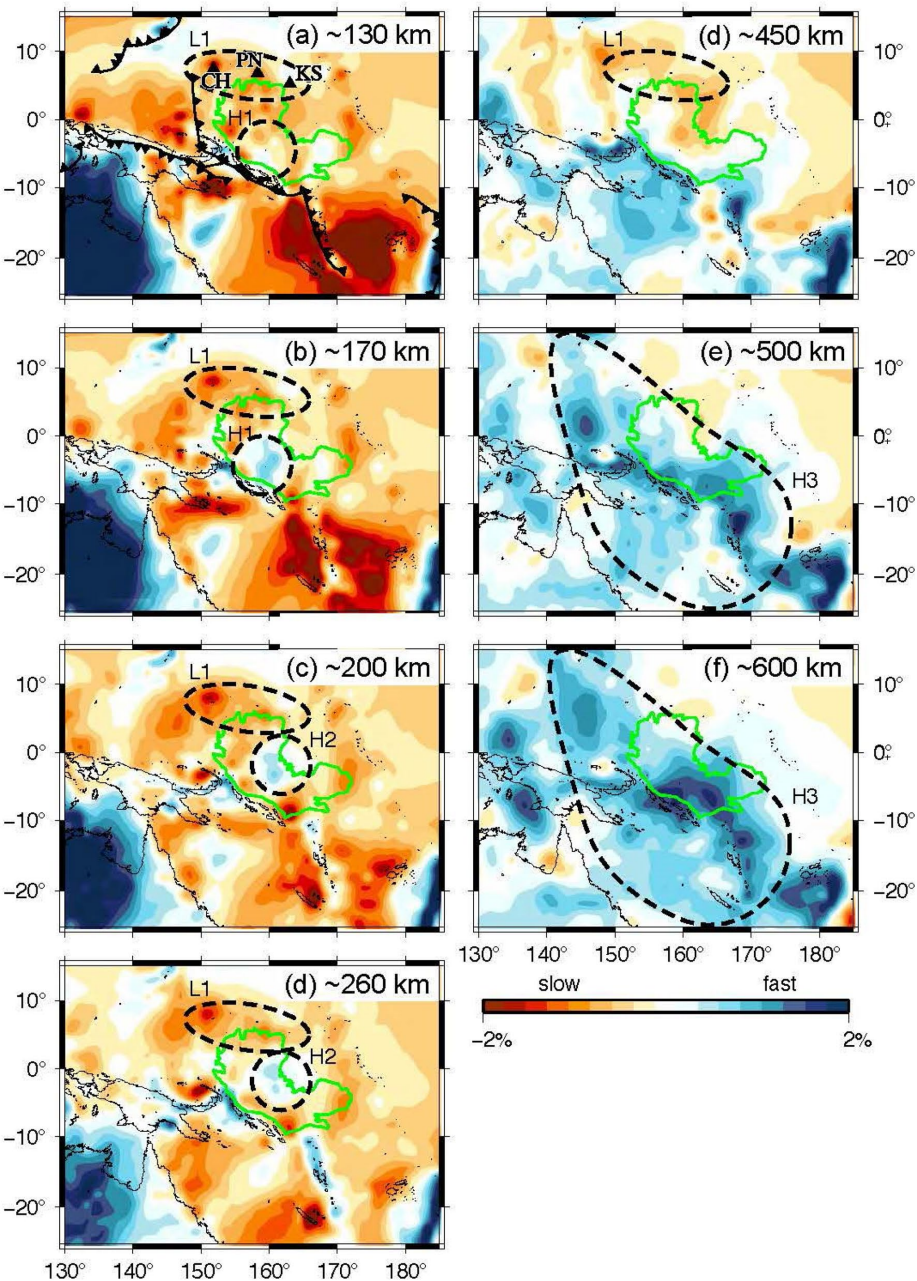
P-wave velocity anomalies at depths of approximately (**a**) 130 km, (**b**) 170 km, (**c**) 200 km, (**d**) 260 km, (**e**) 450 km, (**f**) 500 km, and (**f**) 600 km in the region indicated by thick lines in Fig. 1. The OJP is represented by the green line on each panel. Present subduction boundaries are shown in (**a**). H–H3 and L1 indicate regions of high velocity anomalies and low velocity anomalies discussed in the text, respectively. The GMT 5.4.5^53^ was used to make this figure.

Figure 2 shows P-wave velocity anomalies in and around the OJP at depths ranging from 130 to 600 km. Fast anomalies are observed at the central part of the OJP at a depth of 170 km (region H1 in Fig. 2). At a depth of 130 km, this region exhibits faint velocity anomalies that are faster than the surrounding slow anomaly region by ~ 1%. We consider the fast anomalies in the region H1 at a depth of 130 km to be contaminated by the shallower and surrounding slow anomalies, as the region H1 shows strong slow anomalies at shallower depths (40 km) (Fig. S1) that correspond to the thick OJP crust. The fast anomalies of H1 are similar to recent S-wave velocity models using Rayleigh waves and ambient noises^8^ and the Rayleigh and Love waves of the OJP array^9^. The fast anomalies of the P-wave are imaged deeper than those of the S-wave because the vertical resolution of the P-wave is poor at shallow depths owing to its geometry. A resolution test, in which synthetic fast anomalies of the region H1 at depths of 130 or 170 km depth are reconstructed, shows that the anomalies can be stretched vertically by 30–40 km (Fig. S2). We interpret that the fast S-wave anomalies down to a depth of 150 km associated with the thick lithosphere are elongated vertically in our P-wave velocity image because of the limited vertical resolution at shallow depths.

**Figure 3:**
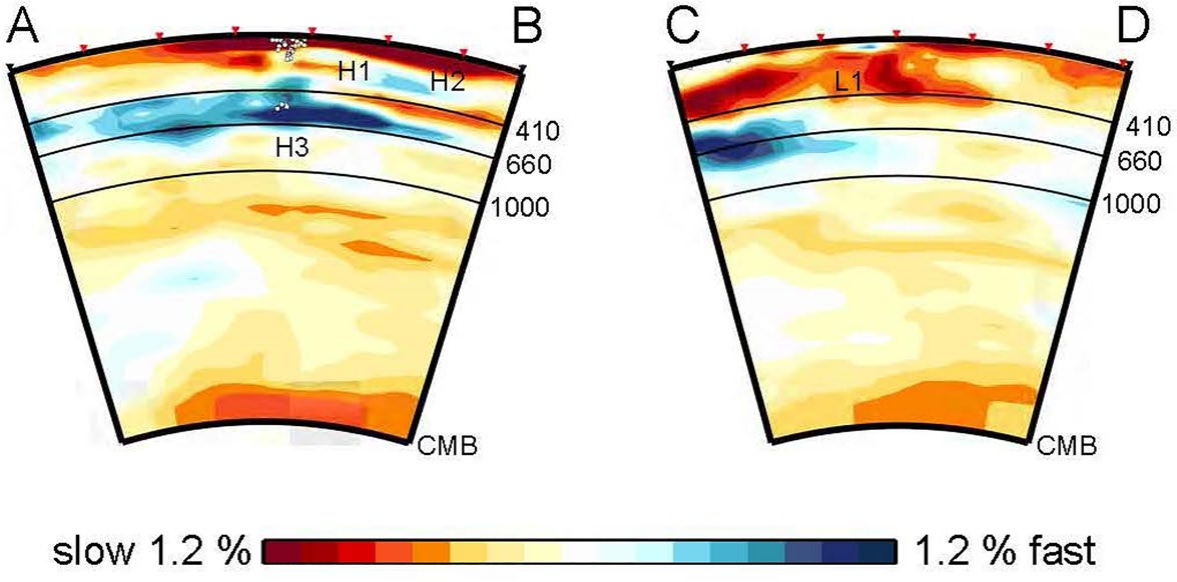
Vertical sections of P-wave velocity anomalies from surface to the core–mantle boundary along the lines AB and CD in Fig. 1. White dots represent hypocenters^54^ within a band that is 50 km wide on both sides of the section plane. Tics are added on the upper frame of each cross section at every 5° from the left hand side. H–H3 and L1 indicate the same anomalous regions in Fig. 2.

Fast anomalies shift northeastward to the edge of the OJP at a depth of 200 km up to 300 km (region H2). Because the smearing of the anomalies in region H1 only occurs vertically and is limited within 30–40 km, H1 and H2 can be considered to be separate fast anomaly regions. The subducting Solomon Sea slab was clearly imaged as fast anomaly region below 200 km while maintaining the cusp at the junction of the San Cristobal and New Britain trenches down to a depth of 450 km. Our model does not show a vertical tear at the cusp as has been suggested by seismicity^27^. The fast anomalies extend in a southwestward direction to the east Australian margin below 450 km. These large fast anomalies toward the southwest of the current subduction boundary correspond to the anomalous area A7 reported by Hall and Spakman^20^ who interpreted it as the Pacific slab subducted from the Melanesian arc that lay horizontally due to the rapid trench retreat between 45 and 25 Ma. Our tomographic image shows that the extensive fast anomalies extend not only in the southwest direction of the subduction boundary but also in the northeast direction to the northeastern margin of the OJP below 500 km (region H3). The cross section across the trench (Fig. 3) shows the spatial relationship between the flat fast anomalies and the current subducting slab indicated by seismicity. The subducting slab is located in the middle of the flat slab. The high Q value below the OJP^28,29^ is considered to be related to the fast anomalies in the mantle transition zone as well as those at shallower depths.

Remarkably slow anomalies are observed at depths of up to 200 km beneath the North Fiji Basin, an active back-arc basin (see e.g., Peltier et al.^30^), which are consistent with the observations of previous studies^20,25^. Slow anomalies exist also at the west (the Caroline plate) and east (the Marshall Islands) of the OJP. The western anomalies beneath the Caroline plate are considered to reflect the young thin Caroline plate and its formation by seafloor spreading during the Oligocene^31^. The eastern anomalies may be related to a small-scale sublithospheric convection along the Marshalls as proposed by Ballmer et al.^32^. Slow anomalies are observed at the northern edge of the OJP above 450 km and extend in the east–west direction (region L1). This region also corresponds to the northeastern margin of the stagnant slab at 600 km depth and the Caroline Islands chain on the surface. Such slow anomalies are observed in S-wave velocity tomography^8,9^. The cross section of the Caroline Islands shows vertical sheet-like slow anomalies extending over ~ 2000 km from the Caroline Ridge in the west to Kosrae in the east.

## Discussion

**Figure 4:**
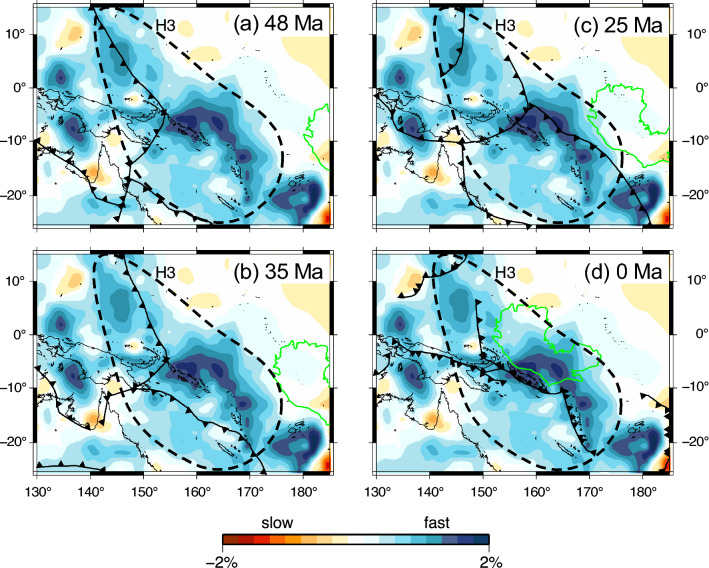
Paleo subduction boundaries and location of the OJP of (**a**) 48 Ma, (**b**) 35 Ma, (**c**) 25 Ma and (**d**) 0 Ma by Seton et al.^16^ superimposed on P-wave velocity anomalies at depth of ~ 600 km. The GMT 5.4.5^53^ was used to make this figure.

We observed flat fast anomalies in the mantle transition zone toward the southwest and northeast of the New Britain and San Cristobal trenches. The fast anomalies connect with near vertically dipping fast anomalies along New Britain and San Cristobal trenches, accordingly they can be interpreted as subducted slabs. The northeastern part of flat slabs beneath the OJP may be intuitively interpreted as stagnant Australian slabs, because they lie in the advancing direction of the Australian plate that currently subducts beneath the Pacific plate. However, it should not be the case: our tomographic image indicates that the total length of the slab reaches to approximately 2000 km including 500 km of the dipping part and 1500 km of the flat part, which is well constrained (Fig. S3). Whereas, the subduction of the Australian plate initiated at most 12 Ma^12–14^, therefore Australian slabs have subducted approximately 1000 km in length to date, assuming a constant subduction rate of 8 cm/year^33^. Yet, the length of the subducted slab observed by the present tomography is as double as that of the Australian slab. Therefore, the fast anomalies beneath the OJP should be attributed to the subducted Pacific slab stagnating in the mantle transition zone. Paleo subduction boundaries at 48, 35, and 25 Ma (Fig. 4) indicate the rapid retreat of the trench where the Pacific plate subducted beneath the Australian plate^16^. The boundary moved over the current boundary from 35 to 25 Ma. The southern edge of the flat fast anomalies ranging within the depths of 450 to 600 km matches the paleo subduction boundary at 48 Ma. The massive stagnant slab from southwest to northeast of the current plate boundary is attributed to the rapid retreat of the subduction boundary. After 25 Ma, as the OJP approached the subduction boundary, the motion at the subduction boundary reversed, and the subduction rate of the Pacific plate decreased accordingly. When the OJP, which has a crustal thickness similar to that of a continent, collided with the trench, the subducting slab could break off from the surface plate^34^. Continued subduction of the Pacific plate accompanied the southward advance of the trench to the present position as indicated by the plate reconstruction model proposed by Hall^15^. Petterson et al.^12^ proposed that soft collision between the OJP and the Solomon island arc at 25 Ma and the subsequent slab breakoff beneath the arc initiated the subduction polarity reversal. The dipping (Pacific) slab detached from the surface plate would have remained beneath the subduction boundary at the time of the break off, and the fast anomalies beneath the middle-eastern edge of OJP at depths of 200 and 300 km (H2) may represent remnant dipping slabs. H2 appears to be disconnected from the stagnant slab as well. It may also be attributable to the reversal of the subduction polarity and trench advance, although the mechanism is not clear.

Sheet-like slow anomalies are observed above the mantle transition zone along the chain of the Caroline Islands of Chuuk, Ponape and Kosrae, which is considered to be a volcanic hotspot chain. They are unlikely to be a smeared image of a plume conduit because our tomography can resolve plume-like slow anomalies near the youngest volcanic island Kosrae (Fig. S4). Such sheet-like anomalies are not typical for the hotspot plume; however, they may be related to the overlap eruptions between Chuuk and Ponape and between Ponape and Kosrae^23^. As an alternative to the hotspot, lithospheric fractures were suggested to induce the lavas in the Caroline Islands^22,35^. Although passive upwelling induced by the lithospheric fractures may form sheet-like slow anomalies, they cannot be rooted in the mantle transition zone as observed in our tomography model. Thus, deep-rooted sheet-like active upwelling most likely causes volcanic chain formation.

Previous tomography studies indicated the slow anomalies of the plume conduit rising through the lower mantle from the bottom of the mantle beneath the Caroline hotspot^36,37^, while it became unclear in the upper mantle. Our tomography also indicates weak slow anomalies from the core–mantle boundary toward the stagnant slab beneath the OJP (Fig. 3). The mantle upwelling conduit that encountered the stagnant slab might spread along the bottom of the stagnant slab and transform into the sheet-like upwelling. Wang^38^ showed an effective elastic thickness of the lithosphere beneath the Caroline Islands that was significantly lower than expected for a normal oceanic lithosphere, indicating that the strength of the lithosphere has been weakened, which may suggest that the lithosphere is heated by the sheet-like thermal anomalies.

Jackson et al.^39^ reported lavas with moderately elevated ^3^He/^4^He ratio from the Caroline volcano chain and attributed this phenomenon to the mixing of deep mantle plume and recycled oceanic crust. This suggests interaction between the stagnant slab and the mantle plume. However, they interpreted that the primitive and recycled domains mix in the deep mantle sources of plumes because lavas with high-^3^He/^4^He are also observed in other locations worldwide such as Hawaii, Galapagos and Samoa.

The Caroline ridge was formed during the eastward passage of the same hotspot from approximately 36 to 18 Ma^31,40–42^. During this period, the magmatism was more intense than that of the Caroline islands. The volumes of the islands decrease as the formation age of the islands decreases from west to east^21^. The stagnation of the slab beneath the OJP was caused by the retreat of the subduction boundary from 35 to 25 Ma. Therefore, the Caroline ridge was formed by the plume conduit directly from the bottom of the mantle with no interference from the stagnant slab. The slab flattened during the interval time between the formation of the Caroline ridge and Chuuk, reducing the magmatism.

The tomographic image suggests an inter-relation among the stagnant Pacific slab, the OJP, and the Caroline volcanic chain: The Pacific slab is stagnant in the mantle transition zone, and a remnant of the subducted Pacific slab, which was broken off from the stagnant slab, is observed in the upper mantle. The Pacific slab was stagnated as a result of the rapid trench retreat from 48 to 25 Ma. The Pacific slab broke off due to the reversal of subduction polarity, which is caused by the collision of the OJP with a subduction boundary. The stagnant slab beneath the OJP may be acting as a barrier against a mantle plume from the lower mantle, which causes a sheet-like upwelling in the upper mantle beneath the Caroline volcanic chain.

## Conclusions

**Figure 5:**
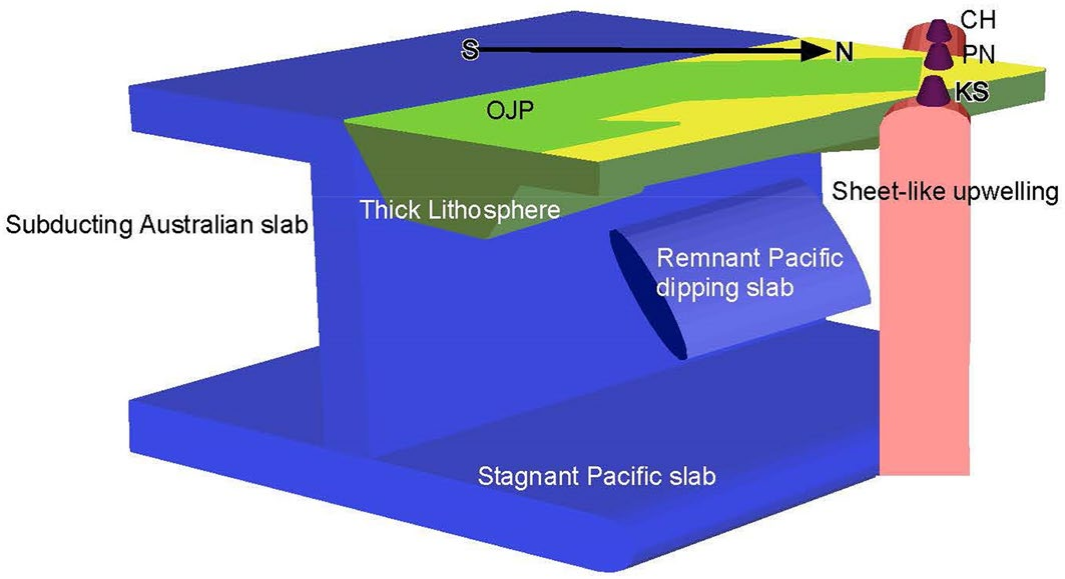
Illustration representing the characteristics observed in in this study: fast anomalies corresponding to the thick lithosphere of the OJP, remnant Pacific dipping slab and stagnant Pacific slab, and sheet-like slow anomalies corresponding to the upwelling along the Caroline volcanic island chain.

We obtained a 3D P-wave velocity structure focusing on the OJP region. Data from the OJP array are effective at improving the resolution around the OJP in the upper mantle. Our findings are summarized as follows and illustrated in Fig. 5.

1. High velocity anomalies were observed underneath the center of the OJP at 130 and 170 km (H1), with an uncertainty of 30–40 km. These fast anomalies are consistent with those of the S-wave anomalies recently obtained^8,9^ and correspond to the thick lithosphere of the OJP.
2. In the mantle transition zone, massive fast anomalies of the stagnant slab were observed from southwest to northeast of the San Cristobal Trench that is the current plate boundary (H3). This broad stagnant slab is associated with the trench retreat from 48 to 25 Ma, where the Pacific plate converged with the Australia plate and retreated to the farthest northeast during 48 and 25 Ma.
3. At depths of 200–300 km, fast anomalies were observed at the middle-eastern edge of the OJP, where the boundary of the Pacific plate subduction retreated to the far northeast at 25 Ma (H2). At the time, the OJP collided with the trench and the subsequent slab breakoff beneath it initiated the subduction polarity reversal^12^. The fast anomalies of H2 is probably the detached dipping slab remaining underneath the boundary at the slab breakoff time.
4. Sheet-like slow anomalies were observed along the Caroline volcanic island chain from the surface down to 450 km depth nevertheless it was considered to be a volcanic hotspot. The slow anomalies are located at a margin of the fast anomalies of the stagnant slabs. The sheet-like anomalies may represent the upwelling which deformed from the mantle plume in the lower mantle because the stagnant slab in between inhibited direct ascent.

## Data and methods

A temporary seismological network “OJP array” was deployed in the OJP and its vicinity from late 2014 to early 2017^26^ (Fig. 1). It consists of 23 broadband ocean-bottom seismic (BBOBS) stations and two land-based broadband stations on Chuuk and Kosrae islands. In Fig. 1, 17 BBOBS stations used in this study are shown. More than 120 events during the 2-years long BBOBS deployment yielded significant measurements. We also collected the seismograms of the island stations of Ponape, Palau and Majuro islands from Pacific21 seismic networks (http://www.jamstec.go.jp/pacific21/ or http://ohpdmc.eri.u-tokyo.ac.jp/) and from stations within the distance of 30° of the center of the OJP via the IRIS (Incorporated Research Institutions for Seismology) data management center. We measured more than 170,000 differential traveltimes as a function of frequency between any two stations by multiband cross-correlation of P waveforms in pass-bands between dominant periods of 30 and 2.7 s, as well as approximately 4400 absolute traveltimes picking. We considered the effects of the reverberation through the different structures of the crusts under the two stations on multi-frequency traveltimes^43,44^. We also measured approximately 500 PP–P differential traveltimes whose PP rays bounced on the surface over the OJP and its vicinity using global stations^45^ (red points in Fig. 1). A correction for the crust under the bounce points^46^ was applied in the PP–P measurement.

By combining these data with approximately 15.8 million first arrival times data from International Seismological Centre catalogue during1964–2014 period, and our original data used in our previous studies; manually picked P-wave traveltimes^47^ and multi-band differential P-wave traveltimes between two stations including BBOBSs in the Northwest Pacific^25,43^ and the French Polynesia region^48^, a 3D P-wave velocity structure was developed for the whole mantle focusing on the OJP. The influence of the structures outside of the region of interest is spontaneously considered by solving for the mantle. For a given differential traveltime between two stations, we obtained a differential travel time kernel by calculating 3D sensitivity kernels for the individual phase arrivals along the two wave paths and subtracting one from the other. Since the sensitivity of phase arrival times to the seismic velocity structure is frequency dependent, we used finite frequency kernels (see e.g., Dahlen et al.^49^) for traveltime data measured at different frequencies. In our tomography, we employed ray-theoretical kernels for the onset times. The inversion using hybrid kernels ensures compatibility while exploiting the different sensitivities of the hand-picked onset times and the differential traveltimes measured by a cross-correlation^50–52^. Obayashi et al.^43^ observed negligible difference between the models obtained using finite frequency kernels and ray-theoretical kernels for onset times.

**Figure 6:**
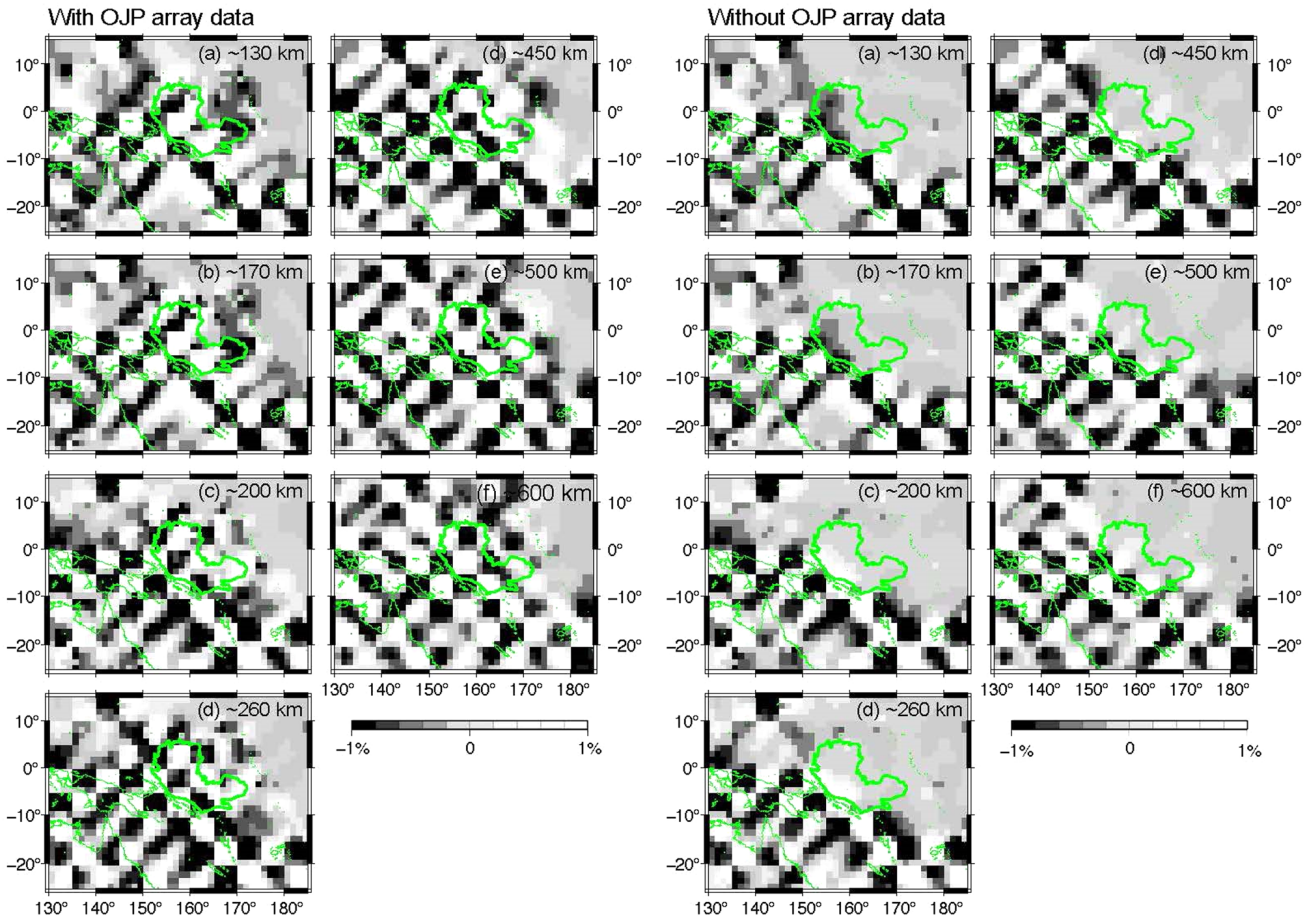
Results of the checkerboard resolution test for the 5° × 5° size pattern in latitude and longitude. (Left) Results based on all the data in this study, (right) results based on all the data excluding data from the OJP array and PP–P differential time data wherein the PP-waves bounce around the OJP. The GMT 5.4.5^53^ was used to make this figure.

The results of checkerboard resolution tests having 5° × 5° alternating positive and negative anomaly indicates that data from the OJP effectively improve the resolution beneath the OJP, and our model has lateral resolution of 600 km through the upper mantle (Fig. 6).

## Supplementary Information

Supplementary Information.

## Data Availability

Seismic waveform data from the OJP Array is available on http://ohpdmc.eri.u-tokyo.ac.jp/dataset/campaign/obs/ojp/index.html and other data used in this study is available at IRIS data management center.
