# Supplementary material for: Interrelation of the stagnant slab, Ontong Java Plateau, and intraplate volcanism as inferred from seismic tomography
Source: Sci Rep. 2021 Oct 28;11:20966. doi: 10.1038/s41598-021-99833-5 (PMC8553740; doi:10.1038/s41598-021-99833-5)
Supplement: Supplementary file 1 — Supplementary Information. [file 41598_2021_99833_MOESM1_ESM.docx]

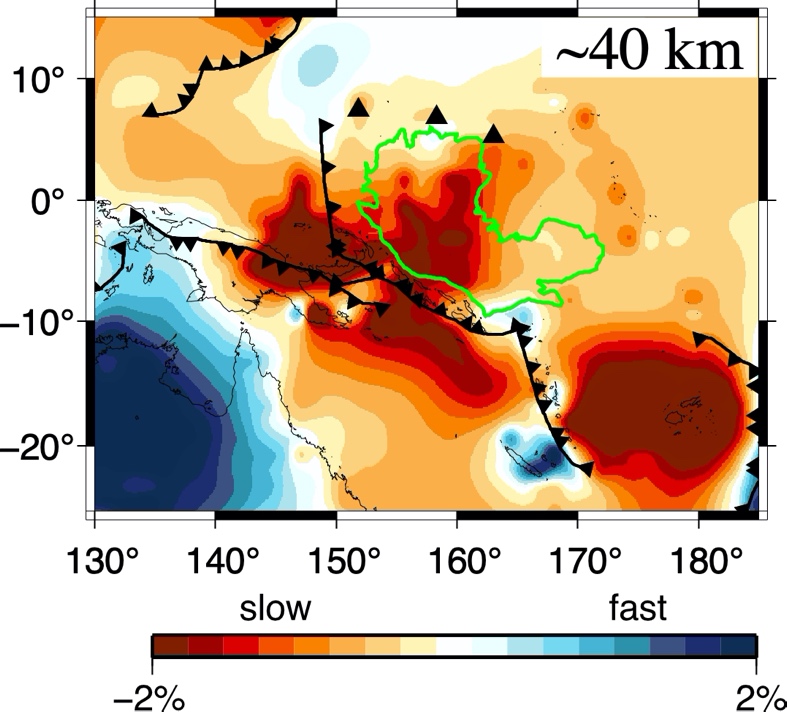


Figure S1. P-wave velocity anomalies at ~40 km depth. Green line indicates the Ontong Java Plateau. Three islands of the Caroline volcanic island chain; Chuuk (CH), Ponape (PN) and Kosrae (KS) are shown by triangles. The low velocity anomaly beneath the center of the OJP.

The Generic Mapping Tools (GMT) 5.4.5 (Wessel et al., 2013) was used to make this figure.


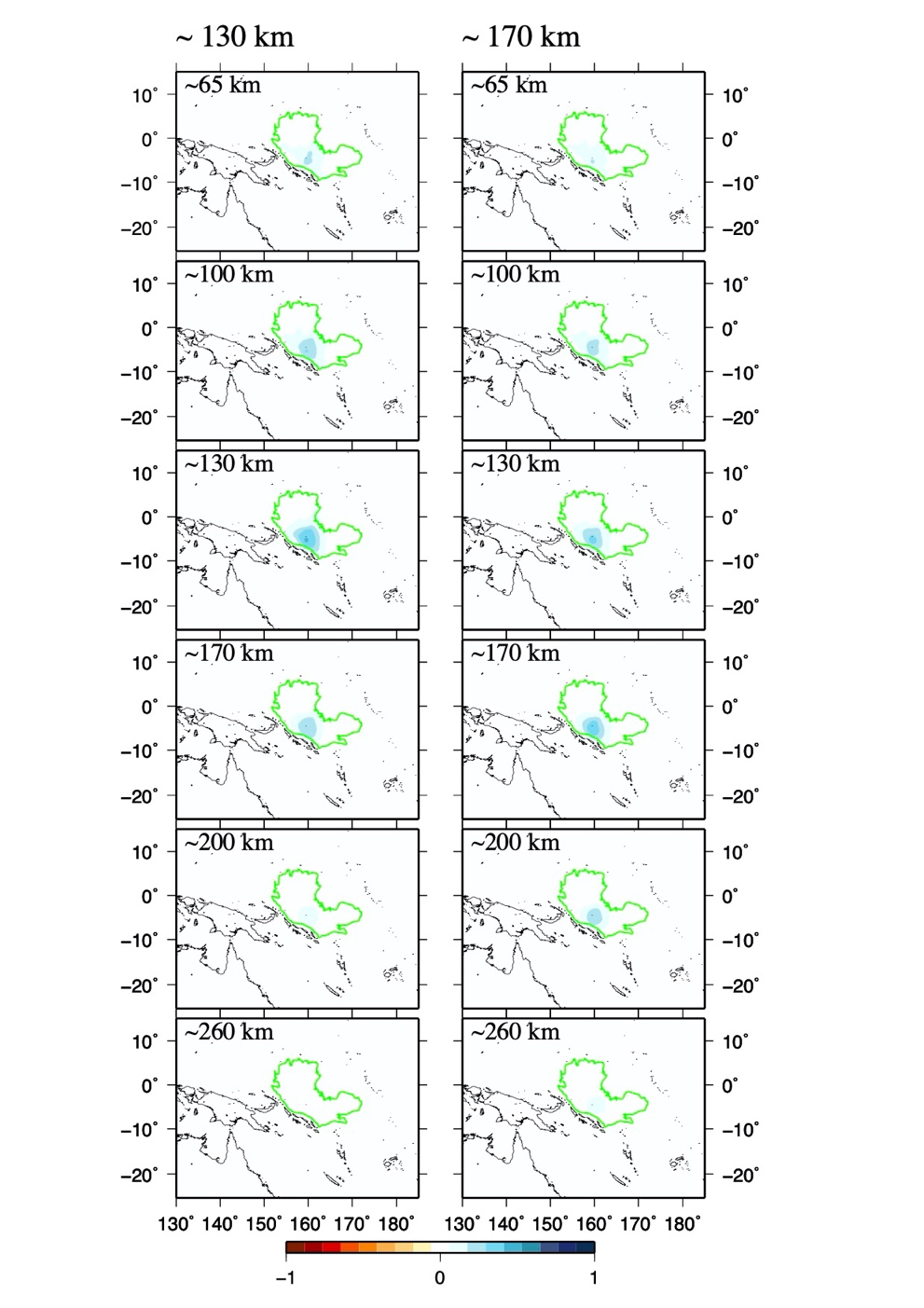


Figure S2

Resolution tests for a 5° sized fast anomaly at the center of the Ontong Java Plateau. 1% fast anomalies at 130 km (left) and 170 km (left) depths. Both of the fast anomalies are recovered with limited lateral smearing. Vertical smearing, however, occurs in both of the shallower and deeper direction within the range of 30–40 km. The GMT 5.4.5 (Wessel et al., 2013) was used to make this figure.


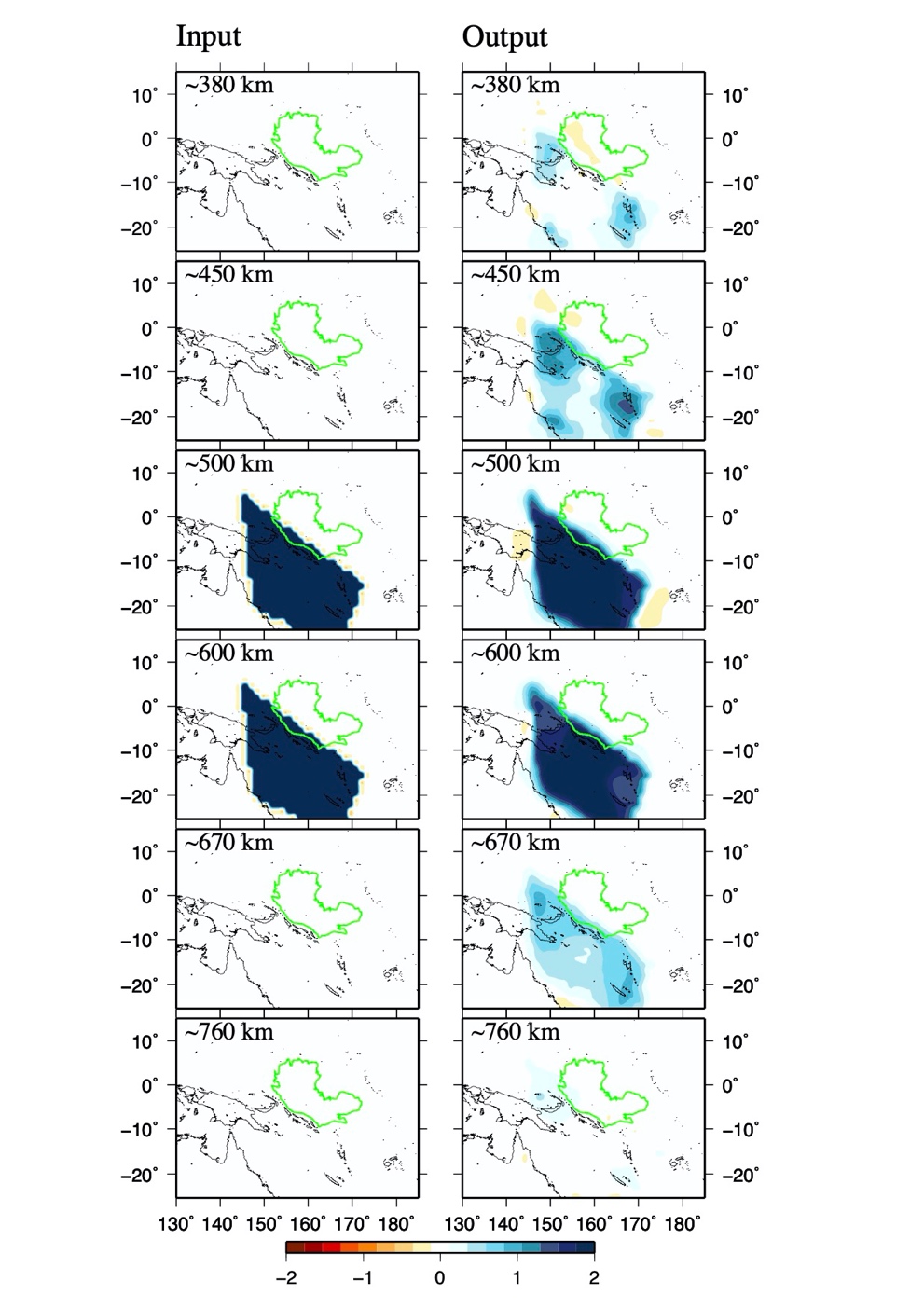


Figure S3

Reconstruction of the input stagnant slab model with 2% fast anomalies. The input model represents our observed massive anomalies expect that northeastern side of the subduction boundary is short. The GMT 5.4.5 (Wessel et al., 2013) was used to make this figure.


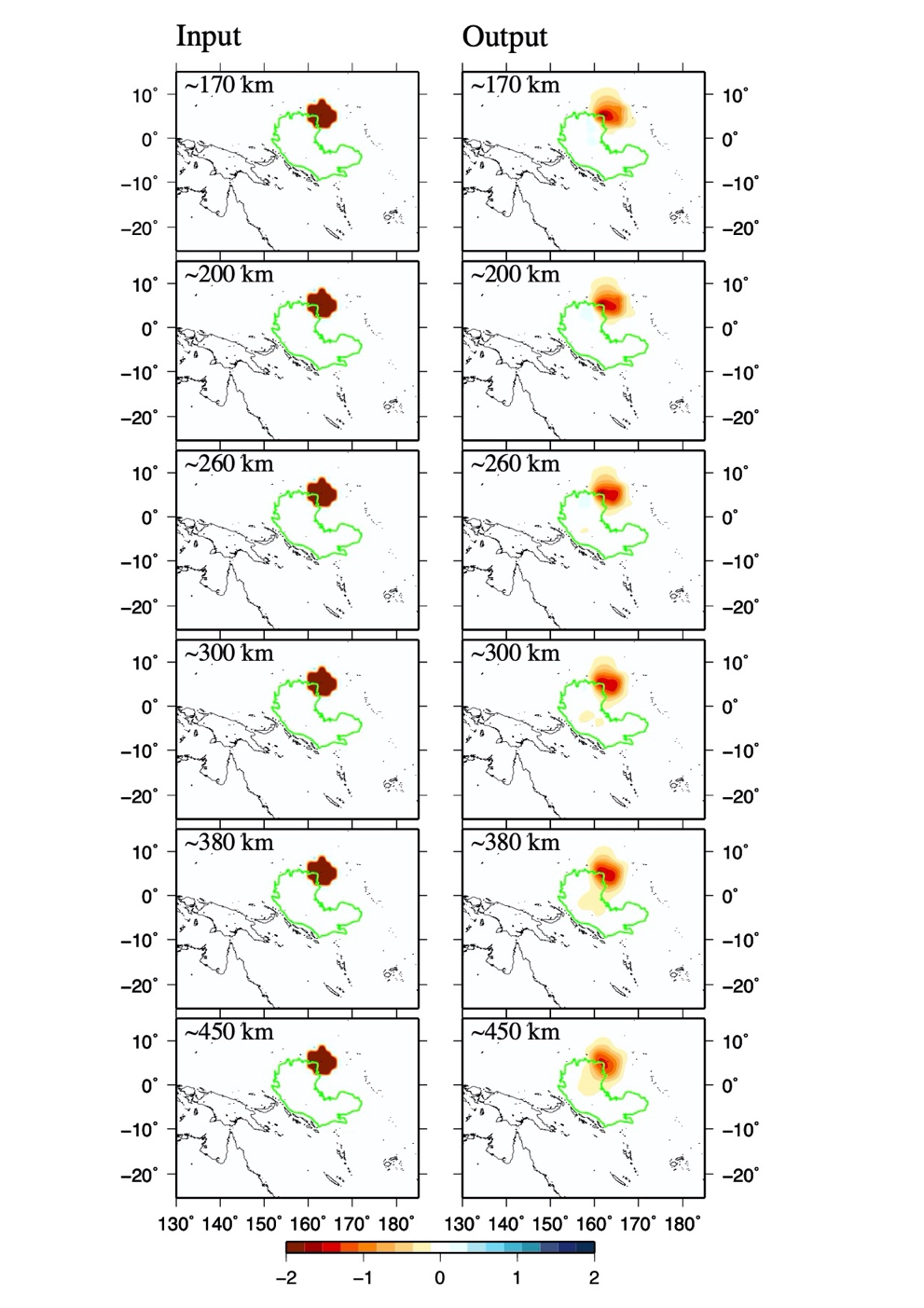


Figure S4

Reconstruction of the input plume-like slow anomalies of 2%. Input model is a slow anomaly column of which diameter is 6° from surface down to 450 km depth near Kosrae. The GMT 5.4.5 (Wessel et al., 2013) was used to make this figure.

Wessel, P., W. H. F. Smith, R. Scharroo, J. Luis, and F. Wobbe, Generic Mapping Tools: Improved Version Released, EOS Trans. AGU, 94(45), p. 409-410, 2013. doi:10.1002/2013EO450001.
